# Post-release behaviour, physiological stress and survival of longline-caught Greenland sharks

**DOI:** 10.1093/conphys/coag031

**Published:** 2026-05-25

**Authors:** Yuuki Y Watanabe, Thomas Stamp, Amanda N Barkley, Kevin J Hedges, Nigel E Hussey

**Affiliations:** Research Center for Integrative Evolutionary Science, The Graduate University for Advanced Studies, SOKENDAI, 1560-35 Kamiyamaguchi, Hayama, Kanagawa 240-0193, Japan; Department of Integrative Biology, University of Windsor, 401 Sunset Avenue, Windsor, ON N9B 3P4, Canada; Department of Integrative Biology, University of Windsor, 401 Sunset Avenue, Windsor, ON N9B 3P4, Canada; Fisheries and Oceans Canada, 501 University Crescent, Winnipeg, MB R3T 2N6, Canada; Department of Integrative Biology, University of Windsor, 401 Sunset Avenue, Windsor, ON N9B 3P4, Canada

**Keywords:** Acoustic tracking, biologging, bycatch, deep-sea fishery, satellite tracking

## Abstract

Incidental capture of sharks by fisheries is common, making it critical to assess species’ physiological and behavioural stress responses and post-release survival. Previous studies have examined blood chemistry, post-release behaviour or long-term survival in various bycatch species; however, few have combined these approaches within a single species. Here, we integrated hook timers, blood chemistry, accelerometry and acoustic and satellite telemetry to assess the impacts of longline capture on Greenland sharks *Somniosus microcephalus*, a deep-sea species encountered in Arctic fisheries. Due to extremely slow growth and late maturity, this species is considered vulnerable to anthropogenic impacts. Lactate and glucose concentrations were within normal ranges for related species and lower than most species. Following release, individuals exhibited reduced vertical movement with elevated swim speed and tailbeat frequency, returning to baseline behaviour after ~ 10 h. Recovery duration was positively correlated with lactate concentration, but not with time hooked. Acoustic and satellite tracking confirmed survival for > 30 days in 9 of 10 tagged individuals, with evidence of multiyear survival up to 6.7 years. The remaining individual moved offshore over 4 days, but long-term survival could not be confirmed. In addition, one shark that was tail-wrapped during capture, for which tracking data were unavailable, may have experienced immediate post-release mortality. Overall, our findings indicate that Greenland sharks are resilient to longline capture stress when properly handled. This resilience likely reflects their slow swim speed and low metabolic rate associated with cold environments, their limited fight response when hooked, and the narrow vertical temperature range of Arctic waters, which minimizes thermal stress during capture. We emphasize the importance of appropriate handling practices and recommend further monitoring of Greenland shark post-release survival from (i) longline fisheries by capture mode (i.e. mouth-hooked, body-tangled or tail-wrapped) and (ii) other fishing gears such as trawl and gillnet.

## Introduction

Sharks and rays (Class Chondrichthyes) are increasingly threatened worldwide, primarily due to direct and indirect effects of fishing (Dulvy *et al*., 2021; Pacoureau *et al*., 2021). In addition to commercial fisheries that directly target sharks, longline fisheries targeting teleosts (e.g. tunas and billfish) frequently catch sharks as bycatch, either dead or alive. Yet, shark bycatch in commercial longline fisheries is often underreported (Campana, 2016), posing a challenge to management and conservation (Oliver *et al*., 2015; Pacoureau *et al*., 2021). A proportion of live shark bycatch is released immediately, either due to low commercial value or in accordance with fishery-specific regulations, such as those established by the International Commission for the Conservation of Atlantic Tunas. Released individuals can nonetheless suffer physical injuries and physiological stress during capture, with potential consequences for post-release survival. Even among survivors, sublethal effects of capture, such as altered blood chemistry and impaired swimming behaviour, may reduce fitness with population-level consequences (Wilson *et al*., 2014). Therefore, understanding the physiological and behavioural stress responses of sharks captured by longline fisheries, and their consequences for survival, is critical for management (Ellis *et al*., 2017).

To address this issue, the stress levels of sharks caught by longlines (or other hook-based fishing gears) are commonly assessed using blood chemistry indicators (e.g. lactate, glucose and pH) (Marshall *et al*., 2012; Prohaska *et al*., 2021), which are then correlated with fishing metrics such as time hooked (Brooks *et al*., 2012; Butcher *et al*., 2015). In addition to blood chemistry, the survival of released individuals can be evaluated over periods ranging from days to months using satellite or acoustic telemetry (Afonso and Hazin, 2014; Gallagher *et al*., 2014; French *et al*., 2015; Sepulveda *et al*., 2015; Mohan *et al*., 2020) with the potential to monitor long-term survival over years. Fine-scale post-release swimming behaviour can also be recorded (typically over several days) using animal-borne accelerometers to assess recovery periods (Whitney *et al*., 2016; Whitney *et al*., 2021; Grainger *et al*., 2022). However, no previous study has integrated all three approaches (blood chemistry analysis, post-release behaviour monitoring and long-term survival evaluation) for a single species to comprehensively assess the impacts of longline fisheries on the fitness and fate of bycaught sharks.

Greenland sharks *Somniosus microcephalus* are large, deep-sea species inhabiting the North Atlantic and Arctic regions (MacNeil *et al*., 2012). Although their current population trend is unknown, their exceptionally slow growth and late age at maturity, with females reaching maturity at ~150 years old (Nielsen *et al*., 2016), suggest that they are particularly vulnerable to anthropogenic impacts. They are a primary bycatch species in commercial longline fisheries targeting Greenland halibut *Reinhardtius hippoglossoides* in the eastern Canadian Arctic and Greenland. While catch rates of Greenland sharks in fisheries are not widely available, two commercial fishing vessels conducting a test Greenland halibut fishery in Cumberland Sound during the summers of 2009 and 2010 captured a total of 659 Greenland sharks (an average of seven individuals per set) over the 2-year period (Madigan *et al*., 2022). Sharks caught alive in halibut fisheries are often severely entangled in longline gear due to their tendency to roll, which complicates their removal. In Canadian waters, both inshore community fisheries and offshore commercial vessels are mandated to release bycaught animals; however, sharks are sometimes killed to facilitate removal from fishing gear (Davis *et al*., 2013). To date, there has been limited research on the impact of fisheries capture on the post-release condition of Greenland sharks. One study reported blood lactate and glucose concentrations of longline-caught individuals (Barkley *et al*., 2017), but post-release recovery and survival have yet to be investigated.

In this study, we adopted an integrated approach to quantify stress levels and their consequences for longline-captured Greenland sharks, combining blood chemistry analyses, accelerometry and acoustic and satellite telemetry. To place our findings in context, we compared our results with published data for other shark species. Greenland sharks are slow swimmers with low metabolic and prey consumption rates, partly due to the cold waters they inhabit (Watanabe *et al*., 2012; Ste-Marie *et al*., 2020; Ste-Marie *et al*., 2022). They also exhibit minimal fight response when captured, despite frequent rolling movements. In general, highly active species that exhibit strong fight responses, such as great hammerhead sharks *Sphyrna mokkaran*, are more vulnerable to capture-related stress (Marshall *et al*., 2012; Gallagher *et al*., 2014). Therefore, we hypothesized that Greenland sharks would be relatively resilient to capture stress associated with longline fisheries. If so, proper handling and release practices in fisheries could result in high post-release survival, thereby mitigating the impacts of bycatch.

## Materials and methods

### Fieldwork

**Figure 1:**
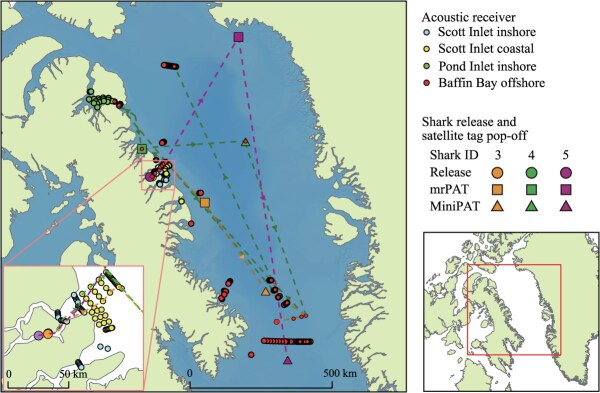
Map of the study area. Greenland sharks were captured, tagged, and released in Scott Inlet (inset, lower left). The locations of the four acoustic receiver arrays (shown in different colours), as well as release sites and satellite tag (mrPAT and MiniPAT) pop-off locations for three individuals (indicated by different colours), are shown. Movement pathways, based on acoustic telemetry detections and satellite tag pop-off locations, are represented by colour-coded dotted lines

This study was conducted with the approval of the University of Windsor Animal Care Committee. Fieldwork was carried out in Scott Inlet (N71° 08′, W72° 08′), a deepwater fjord system located in northeastern Baffin Island, Canada, during the ice-free period of September 2015 and 2016 (Fig. 1). Scott Inlet is the location of a developing commercial longline fishery based in the community of Clyde River, targeting Greenland halibut during the winter months. Longlines were deployed from research vessels (Nuliajuk and Kiviuq II) at depths of 500–900 m and soaked for ~12 h. Each longline consisted of a standard baseline rope (735 m long) with 50 wire gangions (50 cm in length), each equipped with a circle hook (size 16/0 or 18/0). Hook timers (Lindgren-Pitman HT 600) were attached to each gangion. These timers were designed to activate when a magnet was dislodged as a shark pulled on the hook, thereby recording the duration of hook engagement. The study was designed to replicate longline fishing practices used by community fisheries, with the exceptions of using wire rather than braided gangions and employing wider spacing between gangions (Grant *et al*., 2018; Madigan *et al*., 2022). Although coastal fisheries in the eastern Canadian Arctic and Greenland traditionally operate through the ice in winter (Tanaka *et al*., 2025), the development of open water fishing is underway (Madigan *et al*., 2022) and is already prevalent in coastal Greenland waters. A total of 12 sharks were caught, disentangled from the gear and restrained alongside a small vessel using body straps (Table 1). Morphological measurements (e.g. total length) were recorded, and sex was determined. Most individuals appeared in good condition upon capture; however, Shark #11 had severe compression of the caudal trunk region due to entanglement in the gear. To facilitate rapid release, only the accelerometer package was attached to this individual, and no blood sampling or acoustic or satellite tagging was conducted. The hook timers functioned in most cases, but the magnet was not dislodged for Shark #12 (the smallest individual).

**Table 1:** Descriptive information and summary results for longline-captured Greenland sharks

Shark ID	Sex	Total length (cm)	Deployment month/year	Accelerometer recording duration (h)	Lactate (mmol L^-1^)	Glucose (mmol L^-1^)	Time hooked (h)	Recovery duration (h)			Acoustic tracking duration (days)	Satellite tracking
								Diving	Swim speed	Tailbeat frequency		
1	M	256	9/2015	22.3	3.7	5.2	3.3	9.2	8.6	10.2	-	No
2	F	300	9/2015	16.8	8.2	6.0	10.3	>16.8	Data too short	Data too short	4	No
3	F	330	9/2015	38.9	3.2	5.2	13.9	0.5	4.5	2.5	1252	Yes
4	M	222	9/2015	38.2	7.2	5.8	14.0	28.8	17.8	14.5	2456	Yes
5	M	300	9/2015	100.5	5.8	5.6	4.7	13.1	5.1	5	7	Yes
6	M	178	9/2015	12.7	1.3	5.8	1.8	>12.7	Data too short	Data too short	32	No
7	M	286	9/2016	105.6	4.6	5.7	9.6	11.5	17	14.3	1584	No
8	M	312	9/2016	105.9	3.7	5.3	10.6	13.6	11.9	9.1	39	No
9^a^	M	270	9/2016	-	4.1	5.1	9.1	-	-	-	750	No
10	M	223	9/2016	23.7	4.4	6.1	2.4	3.6	3.2	1.6	2068	No
11^b^	F	335	9/2016	-	-	-	-	-	-	-	-	No
12^c^	M	163	9/2016	44.5	2.5	3.1	-	24.2	2.3	1.4	253	No

a ^a^Accelerometer data are missing due to a technical error with the logger.

b ^b^Biologging package did not pop off, no blood sample was collected, and a hook timer was not used.

c ^c^Time hooked is unavailable due to timer malfunction.

Blood samples were collected from the ventral side of the caudal peduncle using a 16-gauge needle attached to a 30-ml syringe. Whole blood lactate and glucose concentrations were measured immediately using portable field devices: Lactate Pro LT-1710 (ARK-RAY) and Accu-Chek Compact Plus glucose meter (Roche Diagnostic). This field method generally produces reliable measurements compared with established laboratory methods (Beecham *et al*., 2006).

A biologging package (~300 g) was attached to the head of each shark by shallowly piercing the skin with a metal needle, passing a plastic cable through the hole, and securing the package in place. The package was programmed to detach from the animal 1–5 days after deployment using a time-scheduled release mechanism (Little Leonardo), float to the surface, send signals via satellite (Wildlife Computers) and VHF transmitters (Advanced Telemetry Systems), and be recovered by boat (Watanabe *et al*., 2004). The package included a W1000- or W2000-PD3GT accelerometer (Little Leonardo), which recorded depth and temperature at 1-s intervals, swim speed (measured using a propeller sensor) at 2–4-s intervals (depending on the individual) and triaxial acceleration at 16 Hz. A technical error occurred with the accelerometer attached to Shark #9, and no data were available for this individual.

In addition, acoustic tags (V16-6H; Innovasea) were surgically implanted in 10 individuals (Table 1). A 5-cm incision was made on the ventral surface of each animal, slightly off the midline and posterior to the pelvic fins. Following sterilization with an 80% betadine solution, the tag was inserted into the peritoneal cavity, and the incision was closed using three interrupted sutures (TriCron blue, 4 × 30″ KV-40). Furthermore, three sharks (#3, #4 and #5) were each outfitted with a mark-report satellite tag (mrPAT; Wildlife Computers) and a pop-up archival satellite tag (MiniPAT; Wildlife Computers) (Table 1). mrPATs transmit location data after release, whereas MiniPAT transmit both location data and archived depth and temperature records. These tags were crimped to an attachment plate secured to the dorsal fins (Hussey *et al*., 2018). mrPATs were programmed to release after 21, 21 and 45 days, and MiniPATs after 45, 45 and 365 days for Sharks #3, #4 and #5, respectively. Following all tagging and sampling procedures, the hook was removed from each shark and the individual was released.

Acoustic receiver arrays spanning four distinct spatial regions were used to monitor post-release movements (Fig. 1): (i) a series of gates located in the inshore environment of Scott Inlet where the sharks were caught (34 receivers, termed ‘Scott inlet inshore’); (ii) a gridded array of receivers covering the coastal area immediately adjacent to Scott Inlet (44 receivers, termed ‘Scott Inlet coastal’); (iii) a gridded array of receivers deployed within the inshore area of Pond Inlet (26 receivers, termed ‘Pond Inlet inshore’); and (iv) a series of receiver gates positioned along the deepwater banks and fisheries management boundary of Baffin Bay and Davis Strait to monitor basin-scale movements (117 receivers, termed ‘Baffin Bay offshore’). The first two arrays were deployed prior to the study period and provided extensive coverage of the inshore and coastal region where the study took place, while the last two arrays were deployed in September 2017. Detection data were available up until September 2022. Although some modifications to array deployments were made over time (Edwards *et al*., 2022), these had no impacts on our results.

### Data analysis

Data obtained from accelerometers were analysed using the software Igor Pro (WaveMetrics) with the Ethographer extension (Sakamoto *et al*., 2009). Instantaneous tailbeat frequency was calculated from lateral acceleration using the continuous wavelet transformation, identifying the peak frequency. A single tailbeat represents the period required for the caudal fin to move from one extreme lateral position back to the original position. Relative swim speed was measured as the number of propeller rotations per second. To convert these values to actual swim speeds (m s^−1^), a flow-tank calibration experiment was conducted. A PD3GT logger was placed in an experimental tank, and the flow speed was increased from 0 to 1.0 m s^−1^ in 0.1 m s^−1^ increments. Because the swim speeds of Greenland sharks were recorded at various intervals (2, 2.5, 3 and 4 s) depending on the individual, the flow-tank experiment was repeated for three different intervals (1, 2 and 4 s). The relationship between swim speed (*Y*) (m s^−1^) and the number of propeller rotations (*X*) (rev. s^−1^) at an interval of *T* (s) can be approximated by the following equation:


\begin{align*} Y&=(X+8.656\times\ln(T)+6)/(55.5\times T)\\&\quad\ \ \ (N=25\ \textrm{data points}, R^{2}=0.99). \end{align*}

Additionally, the stall speed of the sensors was determined to be 0.22 m s^−1^ at 1- and 2-s intervals, and 0.19 m s^−1^ at 4 s intervals, by varying the flow speed in 0.02 m s^−1^ increments around 0.2 m s^−1^.

**Figure 2:**
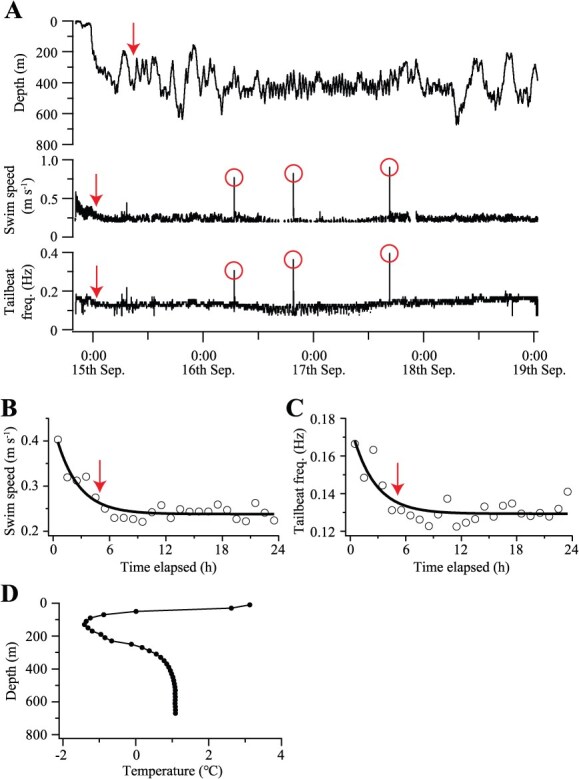
Example of Greenland shark swimming behaviour recorded by an accelerometer (Shark #5). (A) Full record (>4 days) of depth, swim speed and tailbeat frequency. Following release, the shark initially exhibited irregular vertical movements with elevated swim speed and tailbeat frequency. Red arrows indicate the timing at which each parameter was considered stabilized (see text for details). Three burst swimming events are also shown (red circles) following recovery periods. (B) Hourly averaged swim speed and (C) tailbeat frequency during the first 24 h post-release. Data were fitted with negative exponential models to estimate recovery timing (red arrows). (D) Temperature profile in the water column recorded by the accelerometer for this individual

Three types of recovery periods were determined for individual sharks based on (i) diving behaviour, (ii) swim speed and (iii) tailbeat frequency. Regarding (i) diving behaviour, the longest accelerometer records (101–106 h) obtained for Sharks #5, #7 and #8 showed that these individuals exhibited relatively short and irregular diving behaviour (descents and ascents) following an initial descent to depth during the early post-release period (Fig. 2A). Frequency distributions of descent and ascent durations, plotted at 12-h intervals post-release (Fig. S1), revealed that longer descents and ascents (>15 min) began to appear at specific time points post-release for each individual and persisted until the end of the recording period. Accordingly, the recovery period based on diving behaviour was defined as the post-release time when sharks began exhibiting descents or ascents lasting > 15 min (Fig. 2A). Regarding (ii) swim speed and (iii) tailbeat frequency, both parameters were initially high following release and declined over time before stabilizing, indicating a return to normal behaviour (Fig. 2A). To objectively determine when swim speed and tailbeat frequency stabilized, a method similar to a previous study (Whitney *et al*., 2016) was employed. Hourly averaged values (*Y*) for each parameter were plotted against time after release (*X*), and a three-parameter negative exponential model (*Y* = *a* + *b* × exp(−c × *X*)) was fitted (Fig. 2B, C). Recovery duration based on swim speed and tailbeat frequency was defined as the time required for each parameter to reach 80% of the difference between its initial post-release value and its final asymptote value. Because these parameters do not reach their asymptote values by definition, the 80% threshold provides a consistent and practical criterion for calculating recovery duration (Whitney *et al*., 2016). To standardize comparisons across individual sharks, this analysis was limited to the first 24 h of data following release. In addition, the temperature profile of the water column was generated by averaging the water temperature data recorded by the accelerometer within each 20-m-depth bin (i.e. 0–20 m, 20–40 m, etc.; Fig. 2D).

To examine the relationships among blood chemistry (lactate and glucose concentrations), time hooked and behavioural recovery durations (based on diving behaviour, swim speed and tailbeat frequency), a correlation matrix was constructed using Spearman’s rank correlation in RStudio.

For the acoustic tracking data, the number of detections and the amount of time each shark spent within the ‘Scott Inlet inshore’ and ‘Scott Inlet coastal’ areas were examined for the release years (2015 and 2016). Subsequent multiyear detections were identified and classified by all array regions, and total number of detections per day and overall tracking durations were examined as measures of mobility and long-term survival.

## Results

Of the 12 sharks captured, we obtained complete datasets on blood chemistry, time hooked and post-release behaviour from accelerometers (duration: 13–106 h) for nine individuals (Table 1). The biologging package attached to Shark #11, for which blood sampling and acoustic or satellite tracking were not conducted, did not pop off. We speculate that this individual may have died shortly after release and sank to the seafloor, with the absence of body movements preventing release of the package.

Mean lactate and glucose concentrations (*N* = 11) were 4.4 mmol L^−1^ (range: 1.3–8.2 mmol L^−1^) and 5.4 mmol L^−1^ (range: 3.1–6.1 mmol L^−1^), respectively. The mean recovery duration based on diving behaviour (*N* = 8) was 13.1 h (range: 0.5–28.8 h), excluding Sharks #2 and #6, which did not exhibit recovery during their short deployments (13–17 h; Table 1). Seven individuals displayed 1–3 burst swimming events (top-speed range: 0.35–1.02 m s^−1^) (Fig. 2A). Of the 10 burst events recorded, 9 occurred after sharks had resumed what was defined as regular diving behaviour. Based on the decline in swim speed and tailbeat frequency following release (Fig. 2B, C), mean recovery durations (*N* = 8) were 8.8 h (range: 2.3–17.8 h) for swim speed and 7.3 h (range: 1.4–14.5 h) for tailbeat frequency. Sharks #2 and #6 were again excluded from these estimates due to insufficient data length (Table 1).

**Figure 3:**
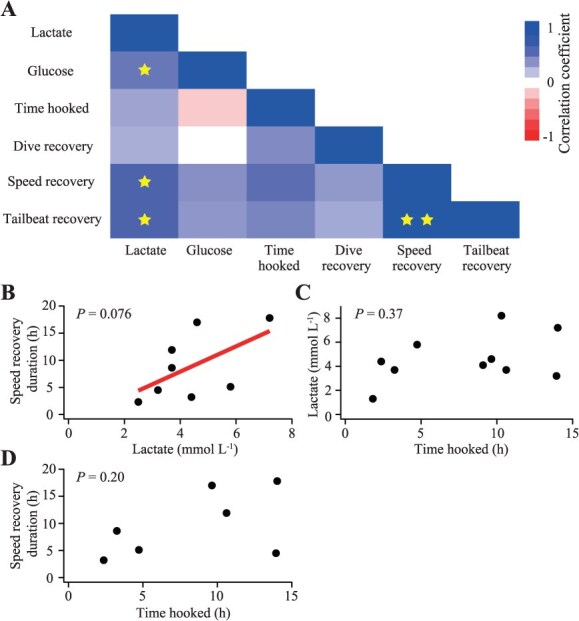
Correlations among blood chemistry (lactate and glucose concentrations), time hooked and behavioural recovery durations (based on diving behaviour, swim speed and tailbeat frequency). (A) Correlation matrix based on Spearman’s rank correlation, with blue and red indicating positive and negative relationships, respectively. Stars indicate cells where marginal (*P* < 0.1, one star) or strong (*P* < 0.001, two stars) positive correlations were detected. (B–D) Representative relationships showing (B) lactate concentration versus recovery duration based on swim speed (red line indicates linear fit), (C) time hooked versus lactate concentration and (D) time hooked versus recovery duration based on swim speed

A correlation matrix incorporating blood chemistry, time hooked and behavioural recovery durations (*N* = 7–11, depending on the parameter combination) revealed a marginal positive correlation between lactate concentration and recovery durations based on swim speed and tailbeat frequency (Fig. 3A, B). Notably, time hooked showed no correlation with either blood parameters or behavioural recovery metrics (Fig. 3A, C, D), although this result may reflect the limited sample size.

**Figure 4:**
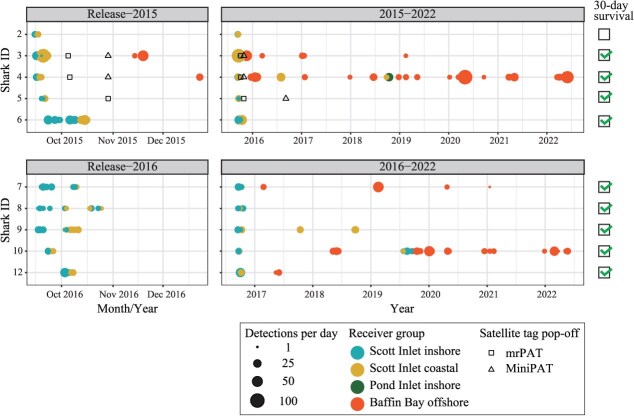
Abacus plot showing acoustic detections of 10 tagged Greenland sharks over time. Points are scaled by the number of detections recorded per day and coloured according to the four acoustic receiver arrays. The left panel displays post-release detections primarily in Scott Inlet (inshore and coastal) for 2015 and 2016, while the right panels show interannual detections. Pop-off dates for satellite tags (mrPAT and MiniPAT) are also indicated. Thirty-day survivals, based on acoustic and satellite telemetry, are denoted by check boxes

All 10 acoustically tagged sharks were detected for periods ranging from 4 days to 6.7 years after tagging and release, with a mean (±SD) tracking duration of 2.3 ± 2.5 years (Table 1). Initially, all tagged sharks were detected on the ‘Scott Inlet inshore’ array for 2–38 (mean: 13.8 ± 12.0) days, with detection counts per individual ranging from 13 to 241 (mean: 78.4 ± 68.5). Sharks then transitioned to the ‘Scott Inlet coastal’ array area, where they remained for an additional 1–37 (mean: 5.8 ± 11.2) days, with detection counts ranging from 21 to 172 (mean: 66.5 ± 61.1) (Fig. 4). One individual (Shark #8) moved intermittently between the inshore and coastal arrays over a 36-day period. All 10 sharks exited the ‘Scott Inlet coastal’ array area, and 5 were subsequently detected on the ‘Baffin Bay offshore’ array. Three individuals (Sharks #4, #9 and #10) were detected returning to Scott Inlet following absences of ~1 to 3 years.

All mrPATs and MiniPAT tags successfully transmitted data, with all locations recorded outside of Scott Inlet (Fig. 1). Combining acoustic and satellite tracking data confirmed survival for at least 30 days in 9 of the 10 sharks (Fig. 4). The remaining individual (Shark #2), which was not equipped with satellite tags, moved from the inshore to coastal array in Scott Inlet within 4 days (confirming survival for at least 4 days) but was not detected by any receivers thereafter.

Overall, there was no evidence of mortality in either the short-term accelerometer data or the long-term acoustic and satellite tag data, with the exception of a potential case for Shark #11.

## Discussion

Despite a relatively small sample size, this study represents the most comprehensive approach to date, integrating blood chemistry analyses, hook timers, accelerometry and acoustic and satellite telemetry to investigate physiological and behavioural stress responses and their consequence for survival in a shark species frequently caught in longline fisheries. This study is also unique in providing the first data on post-release behavioural recovery and survival in Greenland sharks, a species characterized by exceptionally slow growth and late maturity (Nielsen *et al*., 2016) and considered vulnerable if post-capture survival is low.

### Blood chemistry and behavioural recovery

**Figure 5:**
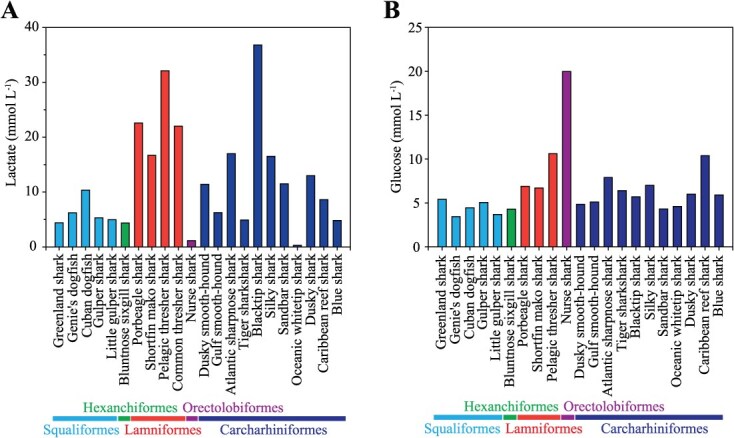
Interspecific comparison of (A) blood lactate and (B) glucose concentrations for longline-captured sharks, with Greenland sharks from this study shown on the far left. Species are coloured by taxonomic orders. See Table S1 for the complete dataset

We measured lactate and glucose concentrations, metabolites commonly used as indicators of physiological stress associated with capture in fishes including elasmobranchs (Marshall *et al*., 2012; Skomal and Mandelman, 2012) in longline-captured Greenland sharks. Lactate is produced through anaerobic glycolysis during exhaustive exercise, such as struggling or fighting when hooked. Glucose concentrations also increase in stressed fishes as gluconeogenesis occurs and hepatic glycogen is mobilized and circulated to metabolically active tissues as blood glucose (Skomal and Mandelman, 2012). Our results (Table 1) are consistent with previous measurements on this species (Barkley *et al*., 2017). We compared our measurements with values reported for other longline-caught shark species from studies that used experimental designs similar to ours (Fig. 5). This comparison should be interpreted with caution, as metabolite concentrations can vary widely within species depending on the individual stress level (e.g. time hooked, body size). Moreover, baseline values remain unclear, and lethal concentrations are highly species specific (Marshall *et al*., 2012; Gallagher *et al*., 2014). Nevertheless, it is apparent that lactate concentrations largely depend on taxonomic order, likely reflecting differences in the tendency to fight when hooked. Greenland sharks are typical among Squaliformes, exhibiting lower concentrations than many species from other orders (Fig. 5A). Glucose concentrations are also generally consistent within an order, but differ among orders, with Greenland sharks exhibiting a low value typical of the Squaliformes (Fig. 5B). We attribute these findings to the species’ lack of a strong fight response when hooked, as well as slow swim speed (Watanabe *et al*., 2012) and low metabolic rates for their size (Ste-Marie *et al*., 2020; Ste-Marie *et al*., 2022), traits associated with the low temperatures of Arctic waters.

The accelerometer records showed that, following release, Greenland sharks initially descended to depth and exhibited limited vertical movements before transitioning to more regularized vertical oscillations within the water column, indicating recovery to normal behaviour. This interpretation is further supported by our observation that sharks displayed burst swimming events, potentially representing prey pursuits, during the latter phase. In pelagic sharks, vertical oscillatory movements are typically associated with prey-search behaviour, although they may also serve other functions such as thermoregulation or navigation (Nakamura *et al*., 2011; Watanabe *et al*., 2021; Braun *et al*., 2022). Thermoregulation is an unlikely explanation for Greenland sharks, as water temperature varied by only a few degrees (−1 to 1°C) across the depth ranges (200–600 m) occupied (Fig. 2D). During the initial ‘disturbed’ phase, both swim speed and tailbeat frequency were elevated, but gradually decreased over time, a pattern previously reported for several shark and teleost species (Sundström and Gruber, 2002; Iosilevskii *et al*., 2022). This trend may reflect post-release individuals actively increasing metabolic rates to oxidate lactate accumulated during capture (Iosilevskii *et al*., 2022) or a ‘fight-or-flight’ response to capture stress mediated by sympathetic activation of the autonomic nervous system (Williams *et al*., 2017).

By quantifying these changes in diving patterns, swim speed and tailbeat frequency, we found that, on average, longline-caught Greenland sharks recovered from capture stress after ~10 h, although considerable inter-individual variation was observed (Table 1). For comparison, behaviour-based recovery durations have been estimated at 6–13 h for many shark species, including blacktip sharks *Carcharhinus limbatus*, white sharks *Carcharodon carcharias*, oceanic whitetip sharks *Carcharhinus longimanus*, and grey reef sharks *C. amblyrhynchos*, among others (Whitney *et al*., 2016; Whitney *et al*., 2021; Grainger *et al*., 2022; Iosilevskii *et al*., 2022). A longer recovery period of around 24 h was reported for lemon sharks *Negaprion acutidens* (Sundström and Gruber, 2002). These comparisons indicate that Greenland sharks are not atypical in terms of behavioural recovery durations following release.

Simultaneous measurements of blood chemistry, time hooked and post-release behaviour allowed an examination of correlation among these parameters. Recovery durations based on swim speed and tailbeat frequency were marginally but positively correlated with blood lactate concentration, supporting the interpretation that post-release periods of elevated swimming activity facilitate lactate clearance (Iosilevskii *et al*., 2022). Unexpectedly, however, neither behavioural recovery duration nor blood metabolite concentrations were correlated with time hooked in this study. In contrast, several previous studies have reported positive relationships between lactate or glucose concentration and time hooked in sharks captured using longlines and other hook-based fishing methods (Brooks *et al*., 2012; Gallagher *et al*., 2014; French *et al*., 2015). Although a larger sample size is needed to draw firm conclusions, our result suggests that, unlike other shark species studied to date, Greenland sharks do not exhibit increasing physiological stress with longer time hooked. This pattern likely reflects their minimal fight response and low metabolic rates associated with cold Arctic waters. Several individuals were hooked for >10 h, during which they may have ceased rolling movements. Notably, Greenland sharks employ buccal-pump ventilation rather than ram ventilation, as observed in more active-swimming species, which allows them to continue breathing while entangled in the gear. Taken together, longline-caught Greenland sharks display relatively minor disturbance on blood chemistry and post-release behaviour compared to more active shark species.

### Horizontal movements and survival

We confirmed that 9 of the 10 (90%) longline-caught Greenland sharks equipped with acoustic tags (and, in some individuals, satellite tags) survived for at least 30 days post-release (Fig. 4). One additional individual (Shark #11), which was not equipped with an acoustic or satellite tag, may have died post-release, as discussed later. The remaining acoustically tagged individual (Shark #2), which did not carry a satellite tag, may also have survived, as it exhibited the expected directional movement from the inshore to coastal array in Scott Inlet within 4 days post-release; however, it was not detected thereafter, and its long-term survival cannot be confirmed. We selected a 30-day threshold based on previous findings that the majority of post-release mortality in sharks occurs shortly after release (Talwar *et al*., 2017; Whitney *et al*., 2021) or within 2 weeks (Gallagher *et al*., 2014; Francis *et al*., 2023). Our results were further supported by detections of five individuals (50%) across the offshore environment (i.e. the ‘Baffin Bay offshore’ array) over extended periods (1–6.7 years), with three sharks returning to Scott Inlet after an absence of at least 1 year. Additionally, sharks equipped with mrPAT and MiniPAT tags displayed extensive movements across Baffin Bay. Together, these findings indicate that all acoustically tagged sharks were active immediately after release within the study area (Scott Inlet) and that most subsequently undertook large-scale movements, consistent with previous finding (Hussey *et al*., 2018; Edwards *et al*., 2022).

When compared with post-release survival studies of other shark species tracked using satellite telemetry, 4-week survival rates were 100%, 74% and 54% for drumline-caught tiger *Galeocerdo cuvier*, bull *Carcharhinus leucas* and great hammerhead sharks, respectively (Gallagher *et al*., 2014). Comparable values for blacktip and shortfin mako sharks *Isurus oxyrinchus* caught in recreational fisheries were 73% and 90%, respectively (French *et al*., 2015; Mohan *et al*., 2020). From short-term (mean 21 h) deployment of accelerometers, variable survival rates ranging from low to high (29% for spinner sharks *Carcharhinus brevipinna*, 58% for blacktip sharks and 93–97% for sandbar *C. plumbeus*, tiger and bull sharks) were reported for longline-caught individuals (Whitney *et al*., 2021). Notably, among deep-sea species similar to Greenland sharks, relatively low short-term (24-h) survival rates of 27–50% have been reported for longline-caught Cuban dogfish *Squalus cubensis* and gulper sharks *Centrophorus* sp., determined by placing individuals in a cage set at the capture depth (Talwar *et al*., 2017). We acknowledge that survival rates can differ markedly depending on capture methods and on whether analyses include only tagged individuals (typically the heathier ones, yielding higher survival rates) or consider the entire fishing interaction—including haulback, handling and release phases—which often produce lower survival estimates (Francis *et al*., 2023). Nevertheless, our comparison demonstrates that Greenland sharks exhibit high post-release survival following longline capture, provided that individuals are disentangled from the gear and handled appropriately.

Similar to blood chemistry and behavioural recovery duration, this species’ tendency not to fight when hooked, combined with its slow swim speed (Watanabe *et al*., 2012) and low metabolic rates (Ste-Marie *et al*., 2020; Ste-Marie *et al*., 2022), likely contribute to high post-release survival. Another important factor is the narrow water temperature range in the water column, which characterizes polar waters (Fig. 2D). The surface temperature (~3°C) was low and only a few degrees above the temperature range (−1 to 1°C) of the sharks’ typical swimming depths, likely inducing minimal thermal stress when the individuals were brought to the surface. In contrast, in temperate and tropical waters, the water column is often thermally stratified, and surface temperatures are much higher than those at depth. The low survival rates of the aforementioned deep-sea shark species can be attributed, at least in part, to exposure to high surface temperature of 30°C—approximately 20°C higher than the temperature at the capture depth (Talwar *et al*., 2017). Even in shallower-swimming species, such as blacktip sharks, elevated surface temperatures are associated with lower post-release survival rates (Whitney *et al*., 2021).

During this experiment, we suspect that only one shark (Shark #11), whose biologging package did not pop off, may have experienced immediate post-release mortality. Unfortunately, its fate could not be confirmed because it was not equipped with acoustic or satellite tags. Upon capture, the caudal trunk of this individual appeared compressed, likely resulting from repeated rolling and entanglement in the longline gear. This indicates that, during retrieval from depth, tension from the gear can cause substantial strain on the caudal region, potentially damaging the vertebral column or associated nerve structures. Consequently, when sharks become entangled around the caudal region, the combination of soft muscle tissue and semi-calcified vertebral cartilage characteristic of this species may increase the risk of post-release mortality. Future studies should quantify the frequency of different capture types (i.e. mouth-hooked, body-tangled or tail-wrapped) and assess their effects on post-release survival.

## Conclusion and implications

Using an integrative approach, we provide support for the hypothesis that Greenland sharks are relatively resilient to longline capture stress. Lactate and glucose concentrations were within the normal range for related species and relatively low compared to other sharks. Following release, individuals initially exhibited reduced vertical movements with elevated swim speed and tailbeat frequency, returning to baseline behaviour after ~ 10 h. Recovery duration was positively related to blood lactate concentration but not to time hooked. Post-release survival derived from acoustic and satellite tracking was high (>90%) during the first 30 days, with clear evidence of long-term survival extending over multiple years. These findings are likely attributable to both biological traits of the species (slow swim speed, low metabolic rate and a limited fight response when captured) and environmental factors (the low and narrow temperature range of the water column).

However, our field observations and previous work (Madigan *et al*., 2022) indicate that achieving the high post-release survival of Greenland sharks observed in this study depends on the implementation of appropriate handling and release procedures by fishers. In community-based coastal fisheries, we propose that sharks be disentangled from the gear or that the wrapped bottom line be cut to facilitate release, with potential incentives provided to offset the time and costs associated with gear repair or replacement. During ice fishing, sharks should be kept in the water as much as possible, rather than dragged onto the ice, to minimize injury. Although our results are not directly transferable to offshore commercial fisheries or other gear types, lifting sharks out of the water by the tail during handling can cause injury and potentially lead to mortality, and should therefore be avoided. Continued research with larger sample sizes is needed to support the sustainable development of both inshore community fisheries and offshore commercial fisheries in the Arctic in the context of bycatch. Future studies should also examine the post-release behaviour and survival of Greenland sharks captured using other fishing gears, such as trawls and gillnets.

## Supplementary Material

Web_Material_coag031

## Data Availability

The data used in Fig. 5 are available in the online supplementary material. Other data underlying this article will be shared by the corresponding author upon reasonable request.
