# Supplementary material for: Post-release behaviour, physiological stress and survival of longline-caught Greenland sharks
Source: Conserv Physiol. 2026 May 25;14(1):coag031. doi: 10.1093/conphys/coag031 (PMC13201077; doi:10.1093/conphys/coag031)
Supplement: Web_Material_coag031 [file web_material_coag031.zip › SuppInfo.pdf]

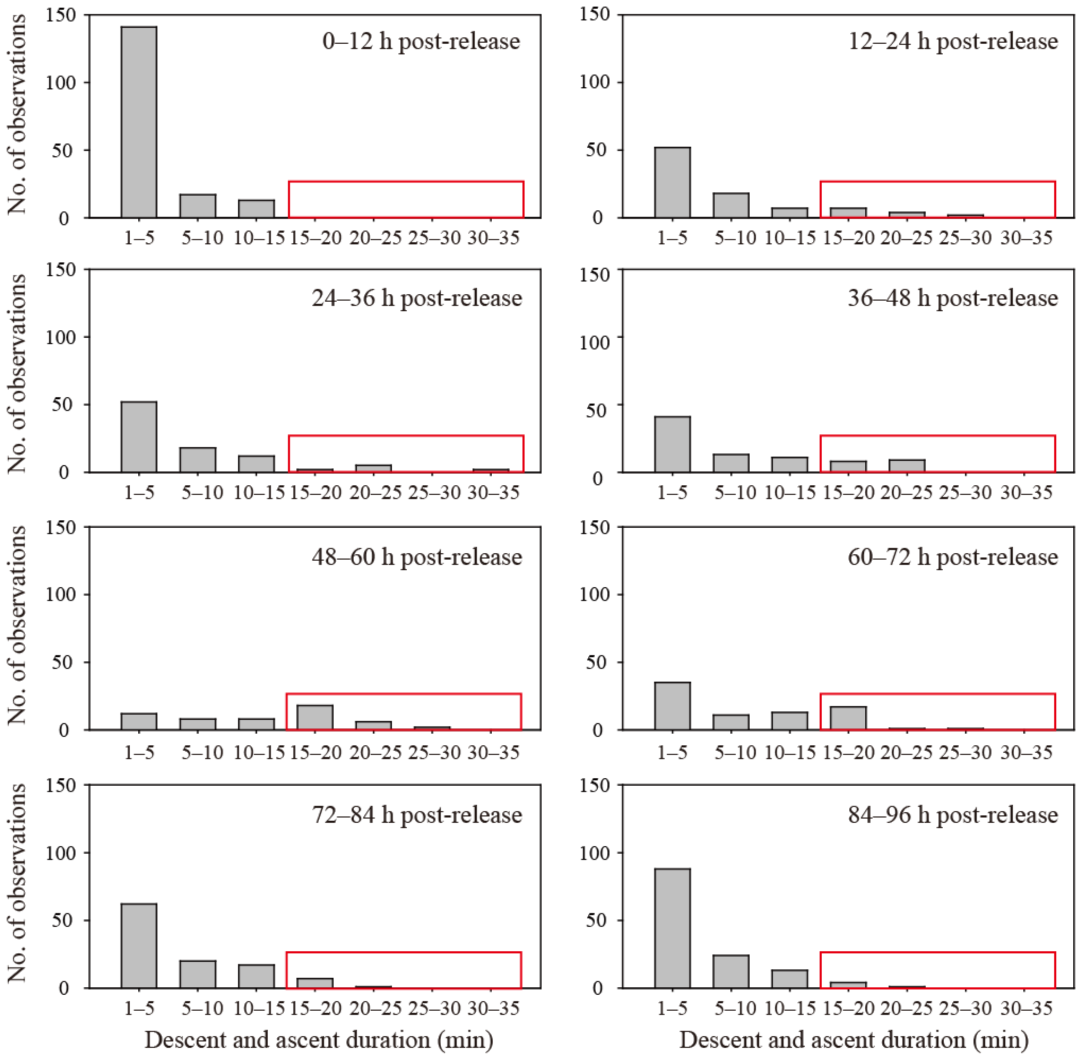

Fig. S1. Frequency distributions of descent and ascent durations plotted for every 12 h post-release for Shark #5, excluding the initial long descent immediately following release. Note that longer descents and ascents (>15 min), highlighted by red rectangular, were initially absent but began to appear 12–24 h post-release and continued until the end of the recording period.

Table S1. Blood lactate and glucose concentrations of longline-captured sharks shown in Fig. 5.

Species	Order	Lactate (mmol L^{-1})	Glucose (mmol L^{-1})	References
Greenland shark (*Somniosus microcephalus*)	Squaliformes	4.4	5.4	This study
Genie's dogfish (*Squalus clarkae*)	Squaliformes	6.22	3.43	(Prohaska et al. 2021)
Cuban dogfish (*Squalus cubensis*)	Squaliformes	10.35	4.45	(Prohaska et al. 2021)
Gulper shark (*Centrophorus granulosus*)	Squaliformes	5.3	5.04	(Prohaska et al. 2021)
Little gulper shark (*Centrophorus uyato*)	Squaliformes	4.97	3.69	(Prohaska et al. 2021)
Bluntnose sixgill shark (*Hexanchus griseus*)	Hexanchiformes	4.35	4.28	(Prohaska et al. 2021)
Porbeagle shark (*Lamna nasus*)	Lamniformes	22.6	6.9	(Marshall et al. 2012)
Shortfin mako shark (*Isurus oxyrinchus*)	Lamniformes	16.7	6.7	(Marshall et al. 2012)
Pelagic thresher shark (*Alopias pelagicus*)	Lamniformes	32.1	10.6	(Marshall et al. 2012)
Common thresher shark (*Alopias vulpinus*)	Lamniformes	22.0	-	(Hight et al. 2007)
Nurse shark (*Ginglymostoma cirratum*)	Orectolobiformes	1.13	19.97	(Bouyoucos et al. 2018)
Dusky smooth-hound (*Mustelus canis*)	Carcharhiniformes	11.4	4.83	(Prohaska et al. 2021)
Gulf smooth-hound (*Mustelus sinusmexicanus*)	Carcharhiniformes	6.25	5.11	(Prohaska et al. 2021)
Atlantic sharpnose shark (*Rhizoprionodon terraenovae*)	Carcharhiniformes	17.0	7.9	(Marshall et al. 2012)
Tiger shark (*Galeocerdo cuvier*)	Carcharhiniformes	4.9	6.4	(Marshall et al. 2012)
Blacktip shark (*Carcharhinus limbatus*)	Carcharhiniformes	36.8	5.7	(Marshall et al. 2012)

Silky shark (*Carcharhinus falciformis*)	Carcharhiniformes	16.5	7.0	(Marshall et al. 2012)
Sandbar shark (*Carcharhinus plumbeus*)	Carcharhiniformes	11.5	4.3	(Marshall et al. 2012)
Oceanic whitetip shark (*Carcharhinus longimanus*)	Carcharhiniformes	0.3	4.6	(Marshall et al. 2012)
Dusky shark (*Carcharhinus obscurus*)	Carcharhiniformes	13.0	6.0	(Marshall et al. 2012)
Caribbean reef shark (*Carcharhinus perezi*)	Carcharhiniformes	8.62	10.39	(Bouyoucos et al. 2018)
Blue shark (*Prionace glauca*)	Carcharhiniformes	4.8	5.9	(Marshall et al. 2012)

References

Bouyoucos, I. A., B. S. Talwar, E. J. Brooks, J. W. Brownscombe, S. J. Cooke, C. D. Suski, and J. W. Mandelman. 2018. Exercise intensity while hooked is associated with physiological status of longline-captured sharks. Conservation Physiology **6**:coy074.

Hight, B. V., D. Holts, J. B. Graham, B. P. Kennedy, V. Taylor, C. A. Sepulveda, D. Bernal, D. Ramon, R. Rasmussen, and N. C. Lai. 2007. Plasma catecholamine levels as indicators of the post-release survivorship of juvenile pelagic sharks caught on experimental drift longlines in the Southern California Bight. Marine and Freshwater Research **58**:145-151.

Marshall, H., L. Field, A. Afiadata, C. Sepulveda, G. Skomal, and D. Bernal. 2012. Hematological indicators of stress in longline-captured sharks. Comparative Biochemistry and Physiology Part A: Molecular & Integrative Physiology **162**:121-129.

Prohaska, B. K., B. S. Talwar, and R. D. Grubbs. 2021. Blood biochemical status of deep-sea sharks following longline capture in the Gulf of Mexico. Conservation Physiology **9**:coaa113.
